# A platform fed-batch process for various CEMAX^®^ producer cell lines

**DOI:** 10.1186/1753-6561-5-S8-P40

**Published:** 2011-11-22

**Authors:** Benedikt Greulich, Marika Poppe, Henry Woischnig, Karlheinz Landauer, Andreas Herrmann

**Affiliations:** 1 Celonic AG, Basel, Switzerland

## 

The random nature of transgene integration harbours various pitfalls for development of production cell lines including clonal variation in expression level and growth characteristics. The CEMAX system is an expression system for targeted integration of expression cassettes via DNA double-strand break induced homologous recombination. Stable high producers are available within 4 weeks without the need of extensive clone screening and process development.

Stable and high expression rates are ensured with the CEMAX system due to targeted integration of a single copy of the gene of interest at a transcriptionally highly active site in the host cell genome. Producer cell lines for various therapeutic product candidates were established. These cell lines produced antibodies and highly glycosylated antibody fusion proteins and showed high clonal similarity. This made a platform fed-batch process profitable.

The CEMAX expression system is based on a genetically modified CHO cell line bearing a tag at a highly active genomic transcription site. The gene of interest (GOI) is integrated via site-directed DNA double-strand break-induced homologous recombination. The target site comprises a screening and selection cassette, which was used for initial development of the high producer host cell. This exchangeable cassette is flanked by elements that facilitate site-directed integration by homologous recombination. These elements include regions of homology for recombination with the CEMAX vector, rudimentary selection markers that are activated during recombination, and cleavage sites for the homing endonuclease I-SceI (Meganuclease).

Transfection of the CEMAX vector comprising the GOI along with transient Meganuclease activity triggers a chain of events after cotransfection: DNA gets cleaved by I-SceI and cellular DNA repair machinery is induced by free DNA double-strand breaks. The CEMAX vector comprising the GOI functions as a repair matrix using recombination between homologous elements flanking the DNA lesion. The GOI gets integrated and the rudimentary selection markers become activated upon homologous recombination. CEMAX producer cells were selected at multi-well plate scale followed by analysis for outgrowth of stable producer cells. The platform process for fed-batch cultivation of various CEMAX producer cells was applied after expansion to the scale needed for production of protein material.

**Figure 1:**
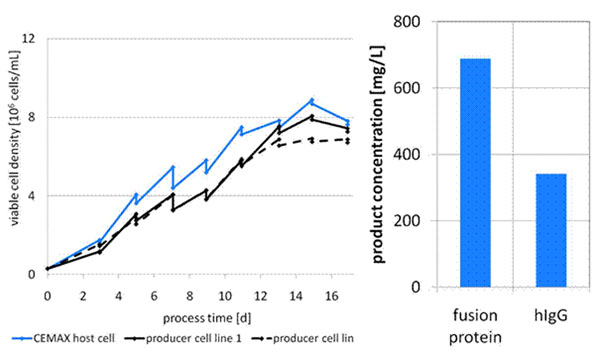
Platform fed-batch process for CEMAX cell lines. Cells were cultivated in comparison to the CEMAX host cell line in shake flask scale. The basal medium was a commercially available chemically defined medium and was used in combination with the proprietary chemically defined feed solution CeloFeed. Product concentration was measured by Protein A HPLC.

The idea of a platform process that is suitable for all CEMAX producer cells was based on theoretical consideration about similarity of cells due to site-directed integration and the observation that cell growth and metabolism of CEMAX cell lines were comparable. This was verified by the results of this study. Applying the fed-batch process cell growth of CEMAX host cells and CEMAX producer cells was comparable (Figure 1). The development of the platform fed-batch process was based on three small scale development steps: (1) basal media screening, (2) feed medium optimization, and (3) improvement of feeding regime. Process development in 1 L bioreactors is currently ongoing.

The fed-batch process comprises a chemically defined and commercially available basal medium and the chemically defined feed solution CeloFeed (Celonic) supplemented according to the improved feeding regime. The glucose level was maintained between 4.5 g/L and 7.5 g/L. Glutamine was kept at a concentration above 0.8 mM. By applying the platform fed-batch process product concentrations up to 690 mg/L were achieved with CEMAX cell lines producing a glycosylated Fc fusion protein after site-directed integration of the GOI (Figure 1). Achievable productivity for different protein products varied from product to product. This was most probably due to different requirements in protein processing and secretion.

The platform fed-batch process was developed by optimization based on commercial available and proprietary basal and feed media formulations. It was suitable for all producer cell lines derived of a particular CEMAX host cell due to highly similar growth behavior after site-directed integration of the gene of interest. The platform process made product concentrations of up to 0.7 g/L achievable. Animal derived component free chemically defined medium and feed solution ensure a robust and regulatory compliant process. By combining the platform process with site-directed integration timelines in cell line development and process development were shortened for faster entry in first-in-man studies.

